# COVID-19 Vaccination Associated Bilateral Pulmonary Embolism: Cause or Coincidence

**DOI:** 10.1155/2022/9596285

**Published:** 2022-09-29

**Authors:** John Omotola Ogunkoya, Marion Itohan Ogunmola, Akinwale Folarin Ogunlade, Akindele Emmanuel Ladele

**Affiliations:** ^1^Benjamin Carson Snr. College of Health and Medical Sciences, Babcock University, Ilishan-Remo, Ogun State, Nigeria; ^2^Division of Respiratory Medicine and Allergy, Department of Medicine, Babcock University Teaching Hospital, Ilishan-Remo, Ogun State, Nigeria; ^3^Family Medicine Department, Babcock University Teaching Hospital, Ilishan-Remo, Ogun State, Nigeria

## Abstract

**Background:**

Acute pulmonary embolism (APE) is a common cause of morbidity and mortality all over the world. Sudden onset dyspnea and chest pain are characteristic. Prior to our index case, only two previous cases of bilateral pulmonary thromboembolism were reported in black Africans and the first to be associated with COVID-19 vaccination. These cases were seen and described in middle-aged men. *Case Summary*. A 59-year-old man presented with a 2 week history of sudden onset dyspnea and a week history of productive cough. No associated chest pain or hemoptysis. No preceding history suggestive of leg pain/swelling. The patient had the booster dose of moderna (mRNA) COVID-19 vaccine a month before the onset of symptoms. There was associated anorexia, generalized body pain, joint pain, and weakness. He had reduced oxygen saturation at presentation with tachycardia. CTPA showed nearly occlusive right and left pulmonary arteries.

**Conclusion:**

Bilateral acute pulmonary embolism is rare all over the world. Its association with COVID-19 vaccine administration is even rarer. However, the clinical presentations and investigation findings are similar to the descriptions available in the literature for unilateral APE.

## 1. Introduction

Acute pulmonary embolism (APE) is a common cause of morbidity and mortality all over the world **[**1**].** The symptoms and signs of APE are similar to those seen among patients with other respiratory and nonrespiratory diseases, making the diagnosis difficult and establishing an accurate diagnosis is a challenging task especially in developing countries such as Nigeria [1]. Sudden onset dyspnea and chest pain are characteristics [2]. Chest pain often resembles the pain of acute myocardial infarction and may be associated with a significant drop in blood pressure leading to shock, tachycardia, tachypnea, restlessness, pallor, and sweating [1, 2]. Many cases are diagnosed at autopsy [3]; however, a thorough history and examination may improve diagnosis even in resource poor settings [1].

In Africa, the prevalence of APE in medical patients varies between 0.14%-61.5% with a mortality rate of between 40%–61.5% [4] without treatment which can be reduced to between 2-8% if promptly diagnosed and placed on anticoagulants [2]. Most cases of APE affect a branch of an artery in a single lung, but few cases of bilateral APE have been described in the literatures, usually in association with underlying lung pathologies such as pulmonary tuberculosis [5], gastrointestinal diseases such as ulcerative colitis [6], sedentary lifestyle, extreme exercise, and orthopedic surgical procedures such as a knee arthroscopy [4].

The moderna mRNA COVID-19 vaccine has been recommended to individuals 18 years of age or older, with a dose of 0.5 ml 9100 *μ*g), given twice, 4-8 weeks apart [7]. Following the advent of mutant strains of this virus, a booster dose was later recommended for the elderly of 65 years of age and older and immune-compromised individuals at least 4 weeks after the 2^nd^ dose was administered [8]. Immunogenicity lasts for up to 119 days after the first vaccination. It is 94.1% effective in preventing severe acute respiratory syndrome due to COVID-19 [7]. At this moment, millions of people have been and are being vaccinated with the moderna mRNA COVID-19 vaccine worldwide, and there are already publications describing isolated cases of acute pulmonary embolism following administration of the COVID-19 vaccine [9, 10].

## 2. Case Summary

A 59-year-old man presented with a 2 week history of sudden onset dyspnea and a week history of cough. Dyspnea was initially noticed during daily routine activities but progressively to dyspnea at rest. Cough was productive of about 2.5 ml of yellowish sputum per bout. There was no associated chest pain, fever, orthopnea, or paroxysmal nocturnal dyspnea. There was no history of lower limb swelling or calf pain, no significant weight loss, or drenching night sweats. He had had 3 doses of moderna (mRNA 1273) COVID 19 vaccine, with the booster dose taken about a month before the onset of symptoms. There were associated anorexia, generalized body pain, joint pain, and weakness, and was bed bound for weeks. There was no significant past medical history. He does not smoke a cigarette or drink alcohol. No family history of any significant medical conditions.

At presentation, he was conscious and alert, no pale, afebrile, anicteric, and well hydrated with no pitting pedal edema. Oxygen saturation was 87% in room air and 95% with supplemental oxygen via nonrebreathe mask at 5 liter/min. Respiratory rate was 30 cpm, no trachea deviation, and bronchial breath sounds with coarse crepitation were heard in the right middle lung zone. Pulse rate was 123 bpm, regular and full volume, blood pressure was 100/85 mmHg and apex beat was not displaced.

Complete blood count, serum electrolytes, urea and creatinine, lipid profile were essentially normal. ESR (Erythrocyte Sedimentation Rate) was 10 mm/hour and CRP (C - reactive protein) was 78.1 mg/L. Sputum gene expert test was negative for Mycobacterium Tuberculosis and sputum culture yielded no growth.

An initial assessment of atypical community acquired pneumonia was made. He was admitted and commenced on intravenous antibiotics, intravenous fluids, and continued on supplemental oxygen. He was subsequently reviewed by the respiratory and cardiology teams due to lack of significant improvement and worsening dyspnea. He gave a history of a similar event about a year prior.

**Figure 1:**
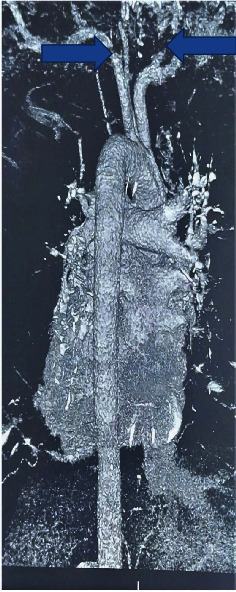
Computed tomography and pulmonary angiography of the chest in the index patient showing near complete occlusion of both right and left pulmonary arteries.

Electrocardiography (ECG) showed sinus tachycardia. Compressive ultrasonography (CUS) of lower limb vessels was essentially normal. Computed tomography pulmonary angiography (ctpa) revealed nearly occlusive pulmonary embolism of the right and left pulmonary arteries with features of possible early pulmonary hypertension. There were also fibrotic streaks on the anterior aspect of the right middle lung lobe and evidence of thoracic spondylosis. (Figure 1) The echocardiogram revealed a dilated right atrium, dilated right ventricle, grade 1 diastolic dysfunction, moderate pulmonary hypertension, and poor right ventricular systolic function with an ejection fraction of 75.18% and minimal pericardial effusion.

An assessment of bilateral pulmonary embolism was made and the patient was moved to the Intensive Care Unit (ICU). He was commenced on Subcutaneous Enoxaparin 80 mg 12 hourly immediately. IV streptokinase 250,000 units was given over 30 minutes and then followed up by 100,000 units after 1 hour. On subsequent reviews, he continued to make sustained clinical improvement. He was moved from the ICU to the medical ward on the 19^th^ day of admission with oxygen saturation of 95-99% on an oxygen concentrator and was commenced on Tab Warfarin 7.5 mg nocte and Tab Sildenafil Citrate 20 mg twice daily. During his admission, he had serial complete blood count, electrolyte, urea and creatinine, and clotting profile tests done. (Table 1) The possibility of a hypercoagulable state was considered, however, protein C and S assays were within normal limits. He was weaned off oxygen on the 28^th^ day of admission.

On the 33^rd^ day of admission, he was discharged home on 7.5 mg of warfarin with oxygen saturation of 96% in room air. He came for follow-up in the chest clinic 2 weeks after discharge. Breathlessness and cough have completely subsided and the patient is alive and well. A repeat thrombophilia workup was not done as the patient could not afford it. Follow-up echocardiography was done with showed a mildly dilated right atrium and ventricle, grade 1 diastolic dysfunction, mild to moderate pulmonary hypertension, and ejection fraction of 81.6%. No evidence of pericardial effusion was seen.

## 3. Discussion

Acute Pulmonary embolism (APE) is a sudden onset partial or complete obstruction to the blood flow of a pulmonary artery or a segment of a pulmonary artery in the lungs by a clot or any substance that move from other parts of the body through the bloodstream (embolism) [11]. Failure to diagnose APE is associated with high mortality and incorrect diagnosis of the condition may unnecessarily expose patients to risks associated with anticoagulant therapy [2]. It often affects a branch or multiple branches of a pulmonary artery but may occasionally affect branches of both pulmonary arteries [11].

Only two other cases of bilateral pulmonary embolism have been reported in black Africans [5, 12] and both were in middle-aged adult males. This case follows that same trend. Although APE is associated with advancing age [2], no strong association has been established between the male sex and APE (unilateral or bilateral). There is, therefore, a need to further explore if any relationship exists between age, the male sex, and bilateral APE in large studies.

Our index patient did not have any calf pain/swelling, a genetic predisposition based on history and available laboratory investigations. However, all patients with a history of thrombotic events should be tested for several inherited conditions, including factor V Leiden, prothrombin gene mutation, protein S deficiency, protein C deficiency, antithrombin deficiency, and fibrin disorders where available [13].

Most patients with APE present with at least one of the four cardinal symptoms of sudden onset dyspnea, chest pain, syncope, and hemoptysis [1, 2, 11]. The index patient, although presented with dyspnea, also had a significant history of productive cough with no hemoptysis. The symptoms of pulmonary embolism are nonspecific. To ensure prompt diagnosis and treatment, a high index of suspicion is needed, especially in patients who are classified as high risk [2]. The occurrence of such symptoms, if not explained otherwise, should alert clinicians to consider PE as a differential diagnosis.

The index patient did not have any preexisting respiratory disease and had no history suggestive of deep vein thrombosis (DVT) but took a booster dose of COVID-19 vaccine a month before presentation. There had been cases of APE in patients within 1-4 weeks of receiving doses of COVID-19 vaccine [9, 10], which is similar to the finding in our index case. However, more needs to be done to determine conclusively the association, if any, between COVID-19 vaccination and hypercoagulability states.

Treatment for APE consists of anticoagulation independent of the etiology of the episode. At diagnosis, patients must receive heparin for immediate anticoagulation, followed by warfarin for maintenance [4]. Duration of warfarin treatment for patients with APE is still a matter of debate; however, the index case was treated with anticoagulants and was continued on oral warfarin for up to 6 months in line with established guidelines [11]. Previous episodes of thrombotic events increase the risk of recurrence, but data regarding such risk in black African and the need for screening and treatment are not available [2].

## 4. Conclusion

In conclusion, we report a case of bilateral pulmonary embolism in a patient with no known risk factors for thrombotic events or previous episode of APE, after the booster dose of the moderna mRNA COVID-19 vaccine. The COVID-19 mRNA vaccine has shown excellent efficacy with a favorable safety profile. Bilateral pulmonary embolism is a rare clinical presentation of APE all over the world and its association with COVID-19 vaccination is even more uncommon However, the clinical presentations and investigation findings are similar to the descriptions available in the literature for unilateral APE.

## Figures and Tables

**Table 1:** The laboratory parameters of the index patient.

Investigations	Values	Reference range
Complete blood count		
a. Total white cell count		4 − 11 × 10^9^/L
Day1	9,400 × 10^9^/L	
Day 7	8,280 × 10^9^/L	
Day 33	5,300 × 10^9^/L	
b. Hemoglobin concentration		36-45 g/dl
Day 1	15.8 g/dl	
Day 7	10.9 g/dl	
Day 33	13.2 g/dl	
c. Platelet count	175,000 × 10^9^/L	250, 000 − 450,000 × 10^9^/L
International normalized ratio (INR)		2-3
Day 2	1.3	
Day 11	1.5	
Day 33	3.0	
Erythrocyte sedimentation rate (ESR)	10	0-22 mm/hour
C- reactive protein	78.1 mg/L	<10 mg/L
Renal function test		
a. Serum urea		1.7-9.1 mmol/L
Day 1	6.5 mmol/L	
Day 7	5.8 mmol/L	
Day 33	3.1 mmol/L	
b. Serum creatinine		53-115 *μ*mol/L
Day 1	163 *μ*mol/L	
Day 7	102 *μ*mol/L	
Day 33	95 *μ*mol/L	
HBA1C	5.0%	<6.5%
Fasting lipid profile		
a. Total cholesterol	4.8 mmol/L	<5.2 mmol/L
b. Triglycerides	1.1 mmol/L	<2 mmol/L
c. HDL-cholesterol	1.6 mmol/L	>1.2 mmol/L
d. LDL-cholesterol	2.8 mmol/L	<3.5 mmol/L
Activated protein C	88 IU/dl	74-112 IU/dl
Protein S	76 IU/dl	70-140 IU/dl

## Data Availability

No data sets were used other than the medical record of the patient.
